# Synthesis of boron nitride nanotubes and their applications

**DOI:** 10.3762/bjnano.6.9

**Published:** 2015-01-08

**Authors:** Saban Kalay, Zehra Yilmaz, Ozlem Sen, Melis Emanet, Emine Kazanc, Mustafa Çulha

**Affiliations:** 1 Department of Genetics and Bioengineering, Yeditepe University, Atasehir, 34755 Istanbul, Turkey

**Keywords:** boron nitride nanotubes, chemical modifications, medical applications, synthesis methods, toxicity

## Abstract

Boron nitride nanotubes (BNNTs) have been increasingly investigated for use in a wide range of applications due to their unique physicochemical properties including high hydrophobicity, heat and electrical insulation, resistance to oxidation, and hydrogen storage capacity. They are also valued for their possible medical and biomedical applications including drug delivery, use in biomaterials, and neutron capture therapy. In this review, BNNT synthesis methods and the surface modification strategies are first discussed, and then their toxicity and application studies are summarized. Finally, a perspective for the future use of these novel materials is discussed.

## Review

### Introduction

Boron nitride nanotubes (BNNTs) are known as structural analogs of carbon nanotubes (CNTs) but with superior properties [1–3]. Although they have structural similarities, they significantly differ in their chemical and physical properties. In contrast to CNTs, their electrical properties are not dependent on their chirality and diameter since they have a large band gap of about 5.5 eV. BNNTs also have excellent radiation shielding properties when compared to CNTs [4]. Since the BNNTs are composed of B and N atoms, their electronic structures are expected to be rather different from that of CNTs. The charge distribution is asymmetric in B–N bonds in BNNTs as compared to the C–C bonds in CNTs [5]. The electron density of B is attracted to the N atoms due to its higher electronegativity. Thus, the B–N bonds have a partially ionic character, which causes a gap between the valence and conduction bands. Therefore, the B–N bonds behave as a wide band gap semiconductor. Some relevant properties of BNNTs are as follows: high hydrophobicity, resistance to oxidation and heat, high hydrogen storage capacity and radiation absorption. Their electrical insulation is indeed very high, despite a high thermal conductivity [6]. Due to these properties, they can be used in a wide range of applications. BNNTs can resist oxidation in air up to 1000 °C while CNTs are resistant only up to 500 °C under the same conditions [7]. This makes BNNTs useful additives to increase stability against the oxidation of surfaces [4]. Due to their highly hydrophobic character, BNNTs were also used to prepare super hydrophobic surfaces [8–9]. A hydrophobic surface was prepared by the synthesis of BNNTs on the surface of a stainless steel substrate where the contact angle was found to be more than 170° [8]. The origin of this super hydrophobicity was attributed to the surface morphology and adsorption capacity of BNNTs for airborne molecules [9].

BNNTs were also used to prepare composite materials to enhance their physical properties. Bansal et al. fabricated a glass composite by adding 4 wt % BNNTs and measured the strength and fracture toughness as 90% and 35%, respectively, which were greater than that of the constituents [10]. BNNTs also have significant hydrogen storage capacity, which was measured as 0.85 wt % – two times larger than that of the commercial CNTs [11].

The use of BNNTs in medical and biomedical applications has also been increasingly investigated [12–14]. Their hydrophobicity and toxicity concerns are the two factors that may limit their use in such applications. Due to their high hydrophobicity, BNNTs can only be used in biological applications after noncovalent [7] or covalent [15–16] modifications to increase their water dispersibility. Thus, they have been modified with several surface modifiers such as PEGylated phospholipids [17], and molecules of biological origin including DNA [18], proteins [13], and flavin mononucleotides (FMN) [19].

The synthesis of BNNTs was first reported in 1995 [20] by Chopra, based on an arc discharge method. Following the first report, several methods including arc discharge [20–22], chemical vapor deposition (CVD) [23–26], substitution reactions [27–29], ball milling [30–35], laser ablation [36–38], and low temperature methods [39–41] were reported. The CVD and ball milling methods are currently the two most widely used methods for the synthesis of BNNTs.

In this review, the most important BNNT synthesis methods are summarized first, then in vitro and in vivo studies of their toxicity are addressed. Finally, the investigations utilizing BNNTs in applications such as drug delivery, biomaterials preparation, biosensors, hydrogen storage, and neutron capture therapy are summarized by giving examples from the literature.

### BNNT synthesis methods

There are several reports on the synthesis of BNNTs. The type of boron precursor, catalyst, temperature, mode of heat and duration are the key parameters in the synthesis procedure. Depending on these conditions, the length and size of the BNNTs will vary. In this section, a summary of the synthesis methods and the nature of the generated BNNTs are discussed. The precursors used in the synthesis of the BNNTs, formation mechanisms; application areas and physical properties (such as diameters and length) are summarized in Table 1.

**Table 1:** Reaction conditions, growth mechanisms and applications of BNNTs reported in literature.^a^

Precursor	*T* [°C]; *t* [h]	Substrate	Method	Growth mechanism	Physical properties	Modification	Application	Ref.

B, h-BN, NH_3_	<1100; 2	iron deposits alumina	ball milling (20 h), CVD	base-growth	40–100 nm diam., bamboo-like	–	–	[42]
	1200; 2				40–100 nm diam., cylindrical shape			

B:FeO:MgO (2:1:1), NH_3_	1200; 0.5	Si/SiO_2_	mechanic. mixed CVD	base-growth	30 nm diam., random direction, closed tip ends	–	–	[43]
	1300; 0.5			tip-growth	60 nm diam., random direction, closed tip ends			
	1400; 0.5			mixed-growth	10 nm diam., flower-like, closed tip ends			

B:FeO:MgO (1:1:1), NH_3_	1300; 0.5			tip-/base- growth	100–500 nm diam., closed tip ends			

B:FeO:MgO (4:1:1), NH_3_	1300; 0.5			tip-/base- growth	50–150 nm diam., closed tip ends			

B_2_O_3_, CaB_6_, Mg, NH_3_	1150; 6	–	CVD	base-growth	150 nm diam., >10 µm length	–	–	[23]

h-BN, N_2_	1250–1300; 10	–	ball milling (100 h), CVD	–	30–60 nm diam., cylindirical shape, 500 nm length	covalent with NH_4_HCO_3_	reinforced material for Al-matrix composite	[44]

B, FeO, MgO	1100–1700; 1	–	ball milling, CVD	metal catalytic growth	50–80 nm diam., up to 10 µm length, straight nanowires	noncoval. polyaniline/ Pt/GO*_X_*	amper. glucose biosensor	[45]

B, iron particle, N_2_	1100; 15	Si/SiO_2_	ball milling (50 h), CVD	metal catalytic growth	50–200 nm diam., up to 1 mm length, bamboo-like	–	insulators for electromechanical systems	[30]

MWCNT, H_3_BO_3_, NH_3_	1300; 3	–	substitution	–	40–50 nm diam.	noncoval. trioctylam., tributylam., triphenyphos.	gel nanocomposite	[46]

B, Co(NO_3_)_2_, N_2_, H_2_	1100; 0.5–3	stainless steel	ball milling, CVD	–	bamboo-like	–	superhydrophobic surface	[8]

B, N_2_	1200; 16	–	ball milling (150 h), CVD	–	20–50 nm diam. cylindrical, cylindrical capped by iron, bamboo-like	–	–	[32]

KBH_4_, NH_4_CI, N_2_	1200–1300; 5–10	–	CVD	–	10–30 nm diam., up to 5 µm length, bamboo-like	–	–	[24]

B, Fe_2_O_3_, NH_3_	1200–1300; 2.25	–	CVD	–	64–136 nm diam., bamboo-like	–	–	[25]

MWCNT, H_3_BO_3_, NH_3_	1080; 6	–	substitution	–	10–100 nm diam., 10 µm length	coval. PVA and HP-MEC	imp. mechanical performance of polymer	[47]

ammon. borane, ferrocn., N_2_	1450; 1	graphite crucible (graphite paper inner line)	CVD	(large diam. catalyst)	300 nm diam., 10 µm length, bamboo-like	–	–	[26]
				vapor–liquid– solid (small diam. catalyst)	15–200 nm diam., 100 µm length, cylindrical shape			

B, Fe_2_O_3_, NH_3_	600; 1	–	CVD	–	20–60 nm diam.	–	hydrogen storage	[11]
B, Fe^3+^-MCM-41, NH_3_					2.5–4 nm diam.			

YB_6_, N_2_/Ar	–	–	arc discharge	mixed-growth	4–10 nm diam., 4–6 µm length, closed or open tip	–	–	[22]

^a^PVA: polyvinyl alcohol, HP-MEC: hydroxypropyl methylcellulose.

#### Arc discharge

BNNTs were first synthesized by an arc discharge method resulting in a 1–3 nm inner diameter and a length of 200 nm [20]. An arc discharge was generated between a hexagonal BN (h-BN)-filled tungsten rod as an anode and a cooled copper electrode as cathode. The dark gray BNNTs were collected from the surface of the copper cathode. Later, hafnium diboride (HfB_2_) electrodes [21], and conductive boron substances such as YB_6_ [22] were used to obtain BNNTs using this arc discharge method.

#### Substitution reaction

Due to the structural similarity between CNTs and BNNTs, BNNTs can be obtained from CNTs via substitution reactions. BNNTs have been synthesized in high yields from CNTs and B_2_O_3_ under a N_2(g)_ atmosphere at 1773 K [27]. A representative substitution reaction is given below.


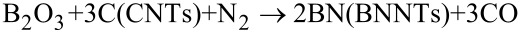


Another substitution reaction was performed by interacting aligned carbon–nitrogen nanotubes (CNxNTs) or CNTs and B_2_O_3_ under NH_3_ atmosphere at 1260 °C for 30 min to synthesize B*_x_*C*_y_*N*_z_*/NTs [28]. The B*_x_*C*_y_*N*_z_*/NTs were obtained from CNxNTs with a higher yield than that of the B*_x_*C*_y_*N*_z_*/NTs obtained from CNTs. Finally, single-walled carbon nanotubes (SWCNTs) were used to obtain multi-walled boron nitride nanotubes (MWBNNTs) by mixing with B_2_O_3_ (as the B precursor) and MoO_3_ (as the catalyst) under N_2(g)_ atmosphere at 1500 °C for 30 min [29]. Although this method can be used to produce BNNTs, the outcome is not always pure BNNTs but rather some B- and N-doped CNTs result in addition [48].

#### Chemical vapor deposition

Chemical vapor deposition (CVD) is a well-known, economical method that is widely used for CNT and BNNT synthesis since it generates high yield products and requires simple experimental procedures. Lourie et al. performed the synthesis of BNNTs from borazine (B_3_N_3_H_6_) based on the reaction provided below [49].


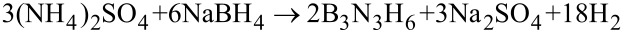


Although Co, Ni, NiB and Ni_2_B were found to be successful catalysts for the synthesis, NiB and Ni_2_B were the most efficient precursors to obtain the highest BNNT yield. Later, Ma et al. demonstrated that BNNTs could be synthesized without any metal catalyst using melamine diborate (C_3_N_6_H_6_∙2H_3_BO_3_) [50]. A comprehensive study also showed that B_3_N_3_H_6_ and decaborane could be used as precursors [51].

In a recent study, a large scale, high-yield BNNT synthesis method was demonstrated based on CVD using boron and metal oxides to produce so-called BOCVD methods [43,52–55]. The chemical mechanism of BOCVD [43] is shown below.


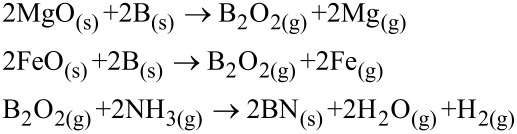


This mechanism is called vapor–liquid–solid (VLS). The high yield of BNNTs was observed as a white powder in the inner wall of the aluminum boat and on the substrate. Note that for the BOCVD mechanism to occur, variation in the types of catalysts, boron compounds and nitrogen-containing gases should be used.

**Figure 1:**
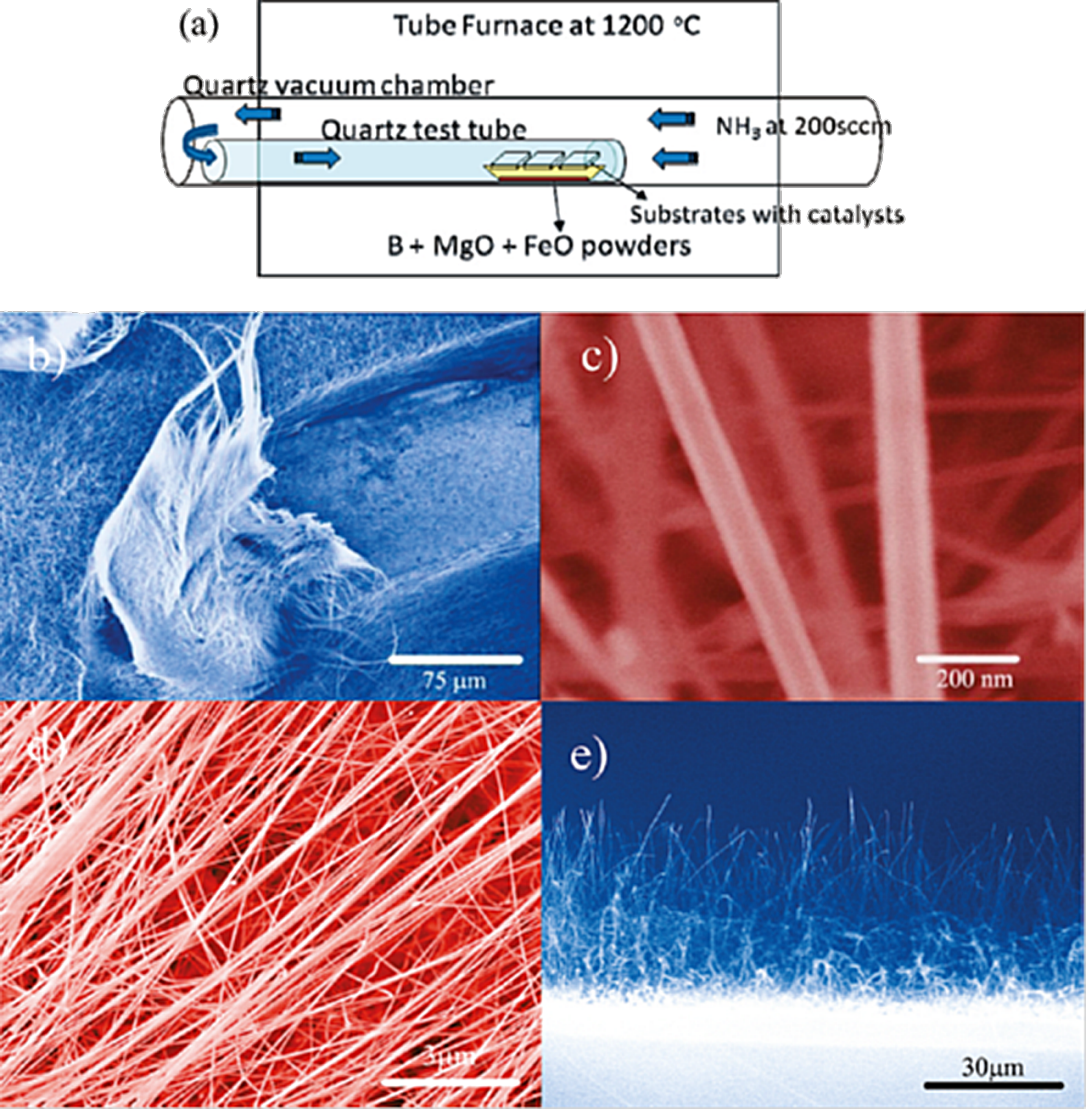
SEM images of BNNTs grown based on a CVD method. (a) Experimental setup, (b) stretching of dense BNNTs from the sample surface, (c) high magnification SEM image of BNNTs, (d) SEM images of slightly compressed BNNTs on a Si substrate, and (e) cross-sectional view of vertically aligned BNNTs. Figure adapted with permission from [56], copyright 2010 American Chemical Society.

The first successful synthesis of patterned BNNTs was performed by catalytic CVD [56]. To produce pure and vertically aligned BNNTs, a Si substrate was coated with Al_2_O_3_ of 30 nm thickness, then MgO, Ni, or Fe catalysts was deposited on the surface of the Al_2_O_3_ by pulsed laser deposition. This substrate was placed into a quartz tube with one end closed. The quartz tube was placed in a tube furnace for the growth of high yield BNNTs at 1100–1200 °C based on the growth vapor trapping (GVT) mechanism as seen in the SEM image in Figure 1.

The latest BOCVD method was studied by Nithya et al. [57]. They claim that large-scale production of BNNTs can be obtained using a mixture of B/V_2_O_5_/Fe_2_O_3_ and B/V_2_O_5_/Ni_2_O_3_ as precursors. In this experiment, the diameter and length of BNNTs was controlled and various BN nanostructures were obtained [57].

**Figure 2:**
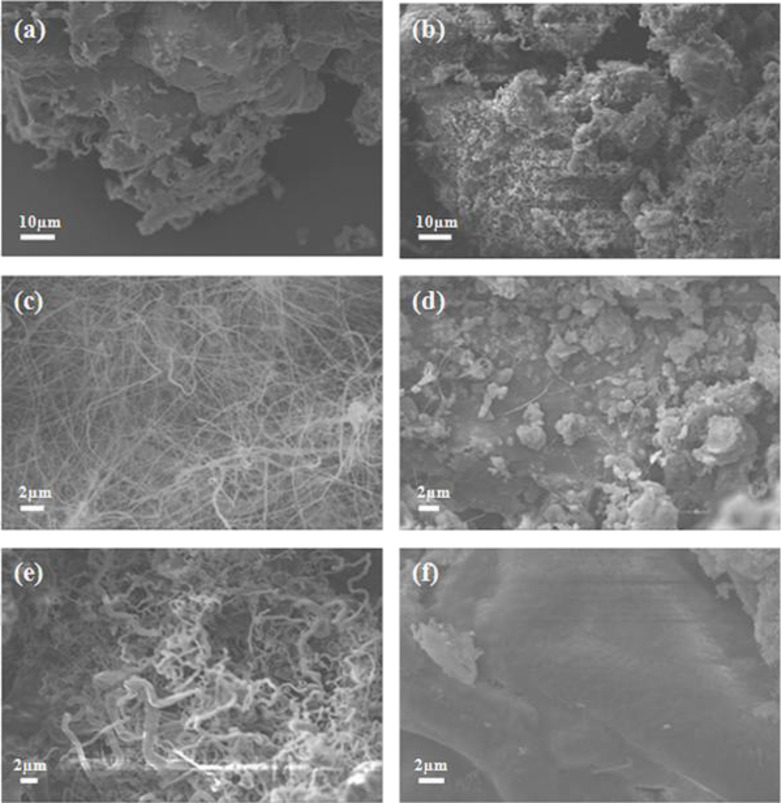
SEM images of the BNNTs products at the different reaction time and colemanite/catalyst ratios (w/w) after CVD application. The respective reaction time and colemanite/catalyst ratio (w/w) were (a) 30 min and 12:1, (b) 60 min and 12:1, (c) 120 min and 12:1, (d) 120 min and 32:1, and (e) 120 min and 8:1. (f) Boat surface after removal of the BNNTs, at 120 min and with a ratio of 12:1.

Recently, our group synthesized BNNTs from a boron ore, colemanite (Ca_6_B_6_O_11_∙5H_2_O), for the first time by means of CVD [58]. The reaction parameters such as type of catalyst, colemanite/catalyst ratio, reaction temperature and duration were optimized. ZnO, Al_2_O_3_, Fe_3_O_4_ and Fe_2_O_3_ catalysts were investigated with respect to their differences in performance. It was found that only Fe_2_O_3_ was effective as a catalyst. Figure 2 shows the SEM images of the results of the BNNT synthesis under several experimental conditions. The synthesized MWBNNTs were in the range of 10–30 nm in diameter, with a 5 nm wall thicknesses and 0.34 nm between walls. This simple method can be used to synthesize pure MWBNNTs on a large scale. The mechanism of BNNT formation was from base-growth, which included the conversion of Fe_2_O_3_ into metallic iron, the formation of an initial complex between metallic iron and BN, and the growth of a BN core into BNNTs on the surface of the metallic iron when the surface was supersaturated with B and N atoms.

In addition to the base-growth mechanism, BNNTs were also formed using the tip-growth, mixed-growth, and metal-catalytic-growth mechanisms (Table 1). During the annealing step in the metal-catalytic-growth mechanism (same as for base-growth), B atoms diffuse into the catalyst particle while the N_2_ is decomposed to N atoms on the surface of the catalyst. The precursors precipitate layer-by-layer to form the BNNTs [30]. In the tip-growth mechanism, the catalyst is located on the tip of growing BNNTs [59]. Thus, the BNNTs are generally formed as bamboo-like structures.

The base- and tip-growth mechanisms of the BNNTs were revealed by TEM showing that the tube diameter and catalysts ratio were the important factors for the selection of growth mechanisms [43]. A ratio of 8:1 colemanite:catalyst caused the formation of BNNTs with a large diameter, thickness and a zigzag structure (Figure 2e) [59]. Under the similar experimental conditions with high catalyst content, the formation of thick BNNTs was reported [43].

#### Ball milling method

Ball milling is commonly used in many studies to obtain a high yield of BNNTs [30]. The main objective of the ball milling method is to increase the surface area to bring the catalyst, boron and nitrogen precursors into contact as much as possible [32]. Although the impurities originating from the steel surface may interfere with the reaction, the synthesis of high-yield BNNTs was claimed [31]. The ball milling method allows transfers a high amount of mechanical energy to the boron powder, which results in an increased surface area and increased number of contact points among the catalyst, boron and nitrogen precursors, resulting in improved yield and product quality [31]. The structural changes in the boron compounds in the reaction mixture during ball milling were observed using X-ray diffraction [33]. It was also reported that there was no chemical reaction between NH_3_ and boron powder during the ball milling process [33]. In the process, the boron precursor is ground either with or without catalyst. However, there is no report comparing both approaches. Li et al. reported that only amorphous boron was ball milled under a NH_3_ atmosphere (300 kPa) for 150 h, at 155 rpm [60]. The milled boron and Fe(NO_3_)_3_ were sonicated in ethanol for 30 min followed by an annealing step under an 85% N_2_ and 15% H_2_ gas mixture. Bamboo-like, 40–80 nm diameter BNNTs were synthesized. Lim et al. reported that amorphous boron powder and NiB*_x_* were ball milled in the presence of NH_3_ atmosphere for 48 h at 180 rpm and annealed in the presence of N_2_ and H_2_ gases [34]. Bamboo-like BNNTs of 20–40 nm diameter and >250 nm length were obtained. Recently, Li et al. reported that the ball milling of amorphous boron powder and Fe(NO_3_)_3_∙9H_2_O is a more effective precursor for a high-yield BNNT synthesis [35]. It was possible to obtain relatively long BNNTs, up to 1 mm in length, using ball milling annealing technique [30]. These long BNNTs were produced over the duration of a 50 h annealing step in the presence of N_2_ gas at 1100 °C.

#### Laser ablation method

The synthesis of single- or double-walled BNNTs can generally be achieved using laser ablation [36–38]. It was reported that the only way to synthesize single-walled BNNTs (SWBNNTs) was by the laser vaporization method [37]. Golberg et al. synthesized pure BNNTs with 3–8 walls from BN under high N_2_ pressure [36]. A single crystalline, 1–20 μm thick BN substrate and a CO_2_ laser with a spot size of 80 µm diameter and 240 W power were used to synthesize pure BNNTs at temperatures higher than 5000 K. Naumov et al. synthesized MWBNNTs using a continuous wave CO_2_ laser at 500 W power based on a BN substrate [61] and Lee et al. obtained gram quantities of SWBNNTs using continuous wave CO_2_ laser [62].

#### BNNT synthesis methods at low temperatures

BNNT synthesis at low temperature is of great interest since it reduces the synthesis cost. However, the temperature has an important effect on the formation mechanism of the BNNTs [42–43]. It has been reported that the BNNT formation mechanism and tube diameter can be varied depending on the temperature or substrate/catalyst ratio [42–43]. Bae et al. obtained exclusively bamboo-liked BNNTs when processed at temperatures below 1100 °C and cylindrical-shaped BNNTs at 1200 °C from the ball-milled boron and FeCI_2_∙4H_2_O precursors [42].

At low temperature, Xu et al. produced 60–350 nm diameter MWBNNTs at 450–600 °C with 50% yield [39]. Wang et al. synthesized BNNTs by means of microwave plasma at a temperature lower than 520 °C [40]. In this technique, a 6–100 nm pore size, aluminum oxide template was used along with microwave plasma. The BNNTs were grown on the surface of this template in the presence of B_2_H_6_/Ar and NH_3_/N_2_ at 10^−4^ Pa pressure at 520 °C. The diameter of the synthesized BNNTs was the same as the pore size diameter of the aluminum oxide template. The BNNTs were synthesized in a stainless steel autoclave at 380 °C from amorphous boron, NaN_3_, and CH_3_CN for 14 h. The obtained product was washed with ethanol, dried and a 5% BNNTs yield was calculated [41].

### Modifications

Although the BNNTs have several unique properties, they are highly hydrophobic and difficult to use when an aqueous media is involved. Significant effort has been dedicated to increase the dispersibility of the BNNTs in aqueous media to extend their applicability to a variety of fields including medicine and biomedical applications. Two approaches to alter the surface properties of BNNTs are commonly employed: one is through covalent attachment of a molecule or molecular structure, and the other involves the physical adsorption of a molecular structure or a polymer onto BNNT surfaces. The chemical modification can be achieved through the –OH groups on the B atom and the –NH_2_ groups at the edges or defects of the BNNTs. There are several polymeric structures that can be physical adsorbed onto the BNNTs to generate coated nanotubes or composites. In this section, examples of these two routes are addressed.

#### Chemical modifications

**Figure 3:**
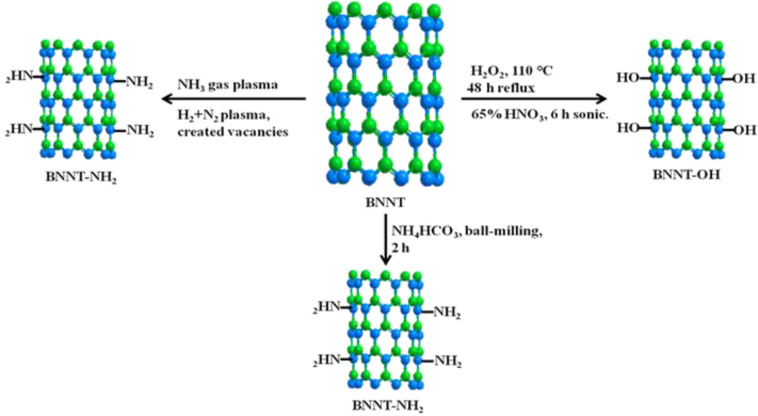
Summary of chemical modification routes of BNNTs.

Due to their high resistance to harsh chemical conditions, BNNTs are consequently difficult materials for covalent functionalization (similar to CNTs). However, recent studies demonstrate that covalent modification is possible. The most commonly preferred chemical functionalization is through the –OH on B atoms and –NH_2_ groups at the edges and defects, as shown in Figure 3 [15,44,63–65].

In one study, BNNTs were functionalized with amine groups by ammonia plasma irradiation [63]. It was predicted that NH_2_∙ radicals produced by NH_3_ plasma attached to B atom at the defects and edges whereas H∙ radicals attached to the N atoms of BNNTs. It was shown the amine-functionalized BNNTs (AF-BNNTs) were dispersible in chloroform. To investigate further chemical functionalization, the AF-BNNTs were coated with 3-bromopropanoyl chloride (BPC) via amide groups of acid chloride of BPC molecules and the amine groups of BNNTs. The study concluded that formation of amine groups on BNNT surfaces was helpful to increase the stability of molecules and polymers on the BNNTs surfaces [63].

**Figure 4:**
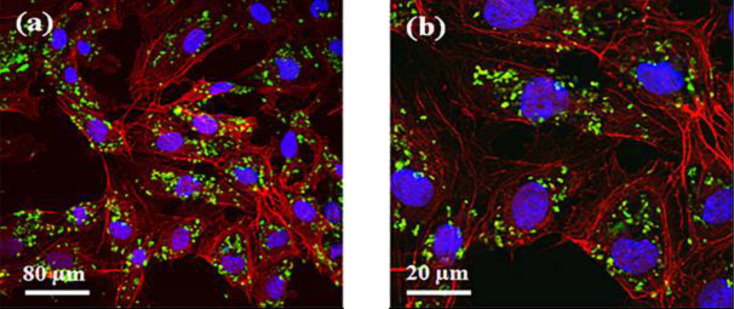
Low (a) and high (b) magnification confocal images of fluorescently labeled, functionalized BNNTs, where red, green, and blue are the cytoskeletal actin, functionalized BNNT, and nuclei, respectively. Figure adapted with permission from [15], copyright 2012 Elsevier.

3-aminopropyltriethoxysilane (APTES) is a widely-employed, aminosilane used in many applications. Ciofani et al. used APTES as an agent for silica coating to functionalize BNNTs [15]. For cellular uptake studies, a fluorescent dye, Oregon Green 488 carboxylic acid, succinimidyl ester was covalently bound to the functionalized BNNTs. The NIH/3T3 fibroblast cells were treated with this fluorescent dye labeled with the functionalized BNNTs. The study found that the labeled BNNTs were localized in the cytosol (Figure 4) [15].

A method to functionalize N–H at the defects on BNNTs with the –COCl groups of stearoyl chloride was successfully implemented [66]. This functionalization increased the dispersibility of the BNNTs in many organic solvents including chloroform, ethanol, acetone, toluene, *N*,*N*-dimethylacetamide, *N*,*N*-dimethylformamide, and tetrahydrofuran. BNNTs functionalized with proteins may increase their potential applications in the field of nanomedicine. The covalent grafting of BNNTs with human transferrin, linked through a carbamide bond, was reported [67]. The transferrin–BNNTs were tested on primary human umbilical vein endothelial cells (HUVECs) to investigate their cellular uptake. It was concluded that the functionalization of the BNNTs via a targeting protein could generate smart and selective nanocarriers to be used in nanomedicine [67].

#### Physical modifications

For these types of modifications, weak interactions such as π–π, hydrophobic, and van der Waals forces are utilized to coat the BNNTs with mostly a polymeric material. Poly[*m*-phenylenevinylene-co-(2,5-dioctoxy-*p*-phenylenevinylene)] (PmPV) was used to cover BNNTs via π–π interactions [7]. The prepared structure was more soluble in chloroform and ethanol than water. It was claimed that this composition had potential use in optical devices because of its luminescence properties and good dispersibility in organic solvents [7].

The BNNTs were also coated with PEGylated phospholipid 1,2-distearoyl-*sn*-glycero-3-phosphoethanolamine-*N*-[methoxy(poly(ethylene glycol))] conjugates (mPEG–DSPE)] [17]. This polymer was selected due to its water solubility and biocompatibility. The mPEG–DSPE/BNNTs suspension was expected to be stable in water because fatty acids from DSPE should noncovalently interact with BNNTs and the hydrophilic mPEG could aid in the dispersion of the BNNTs in water. Indeed, the mPEG–DSPE/BNNTs were highly dispersed in water and slightly so in ethanol, acetone, methanol and chloroform. Furthermore, it was noted that sonication for long time periods (hours) could help to better disperse the BNNTs [17].

FMN is derived from vitamin B_2_ and is a well-known phosphorylated biomolecule. The interaction of vitamin B_2_ with BNNTs resulted in a highly fluorescent FMN–BNNT complex under daylight and UV light irridation [19]. Furthermore, the fluorescence from this complex was thermally stable and pH-dependent. It was suggested that FMN–BNNT nanohybrids could be used for biomedical imaging.

**Figure 5:**
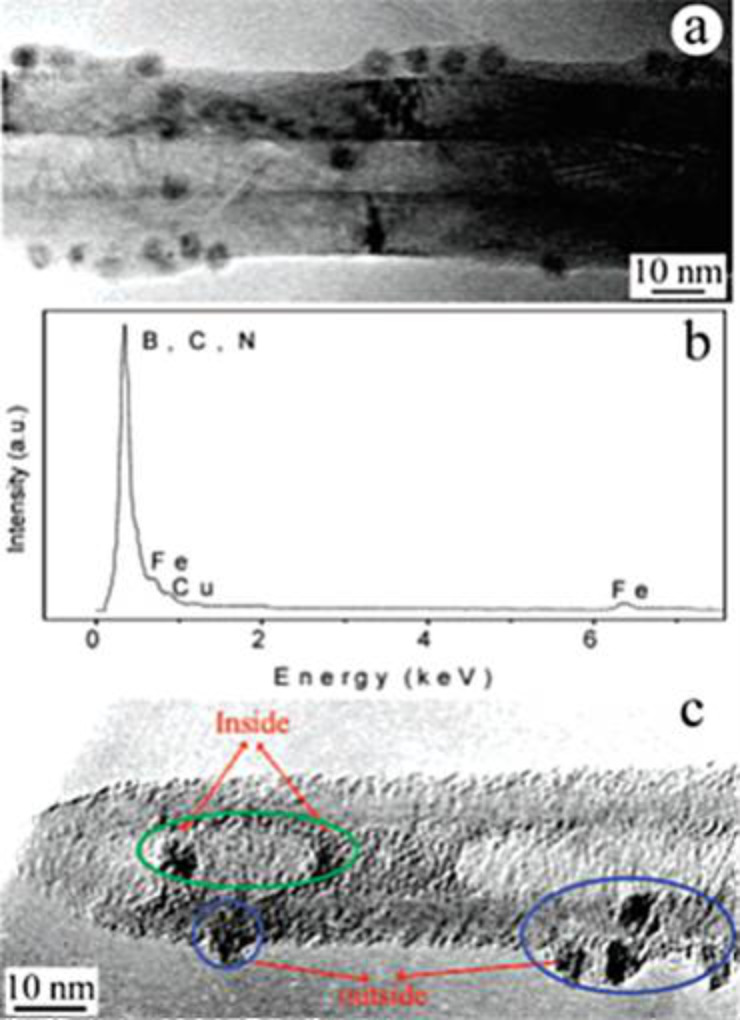
TEM images of ferritin molecules immobilized onto BNNT surfaces (a), EDS spectrum of BNNTs with immobilized ferritin molecules (b), ferritin molecules on the surface and inside of a BNNT (c). Figure adapted with permission from [13], copyright 2005 American Chemical Society.

The adsorption of ferritin onto BNNTs was also reported. It was found that there was a natural affinity of this protein to the BNNT surfaces [13]. Figure 5 shows TEM images of ferritin immobilized onto the BNNTs. As seen, the ferritin not only can be adsorbed onto the BNNT surface but can also penetrate into the BNNTs. In addition, 1-pyrenebutyric acid *N*-hydroxysuccinimide ester (PAHE) was used to obtain more efficient immobilization. Because the PAHE has aromatic pyrenyl groups in its structure, a strong π–π interaction between the BNNT surface and PAHE is expected. When PAHE was used, a denser ferritin immobilization was observed [13].

A computational investigation concerning the interactions of BNNTs with tryptophan (Trp), aspartic acid (Asp), and arginine (Arg) was also carried out [68]. It was found that the polar Asp and Arg interacted with the BNNT surface through the charge transfer and electrostatic interactions while Trp, a neutral amino acid, had no interaction with the surface of the BNNTs. This study provides a deeper understanding into the nature of the interactions of amino acids (and perhaps similar molecules) with surface of the BNNTs [68].

The interaction of a peptide, HWSAWWIRSNQS, with BNNTs was studied using AFM [69]. It was found that the peptide–BNNT structure had an excellent dispersibility in water since the BNNTs were covered by this peptide. The study revealed that the presence of Trp (W), which has a benzene ring in the peptide sequence, exhibited a strong π–π interaction with the BNNT surfaces [69].

Dendrimers prepared from synthetic carbohydrate ligands were used to coat the BNNTs to mimic the cell surface receptors. The [G-2] dendrimers possessing R-mannose moieties ([G-2] Man) [70] were used to coat the BNNTs. Although uncoated BNNTs were precipitated very quickly, the [G-2] Man-coated BNNTs formed a stable suspension in water for weeks. The carbohydrates provide specific molecular recognition sites on cell membranes. The [G-2] Man-coated BNNTs were incubated with the R-mannose-specific receptor *Canavalia ensiformis* agglutinin (Con A). To observe the fluorescence of this complex, they were conjugated with fluorescein isothiocyanate (FITC). Chinese hamster ovary (CHO) cells were treated with this complex. It was found that the coated BNNTs had specific molecular recognition capability [70].

Glycol chitosan (GC) is widely used due to its biocompatibility and good solubility over a broad pH range [71]. The BNNTs were coated with GC during a 12 h sonication process. The TEM results indicated that the GC–BNNTs had two different configurations: bamboo-like shaped and noncontinuous walled. HUVECs were treated with the GC–BNNTs and the cellular uptake of the GC–BNNTs was observed. However, the uptake mechanism remains unclear and it might be worthwhile to further investigate it [71].

BNNT-grafted, poly(glycidyl methacrylate) and polystyrene brushes were prepared via atom transfer radical polymerization [72]. The resulting nanocomposite material was characterized using FTIR, TGA, SEM and TEM. The TEM images clearly show the formation of polymer grafts on the BNNT surface.

### Toxicity of BNNTs

The potential adverse effect of nanomaterials on living systems is a growing concern. Although many engineered nanomaterials (ENMs) are already in use in several applications, there is no clear consensus regarding their possible impacts on living systems and the environment. The main reason behind this uncertainty is the lack of significant data on the subject. In addition, the diversity of nanomaterials and parameters further adds to the uncertainty for the proper assessment of the safety of these novel materials. Similar to many other ENMs, there are several issues with the assessment of the possible toxic effects of BNNTs. In early studies, there was no clear consensus regarding their cytotoxicity. In some reports it was found that BNNTs were toxic [73], and in others, not [74]. Naturally, first, in vitro studies were undertaken to assess the toxicity of the BNNTs. Similar to CNTs, one of the major problems in toxicity assessment of BNNTs is their low dispersibility in aqueous media, due to their high hydrophobicity. In order to increase the dispersion, either a surfactant or hydrophilic polymer is used to alter the surface properties. However, this process adds further uncertainties to the assessment since another material is introduced into the system. For example, polyethylenimine (PEI) is a cytocompatible polymer and principally used for DNA transfection and cell permeabilization. The BNNTs were coated with PEI for dispersion in aqueous media for biological applications [12]. The effect of the PEI-coated BNNTs with respect to viability, metabolism and cell proliferation of human neuroblastoma cell line (SH-SY5Y) was investigated. The PEI-coated BNNTs exposed cells analyzed at different time intervals. The viability, metabolic activity and proliferation of the cells were analyzed with MTT and trypan blue assays. The results showed that the PEI-coated BNNTs did not affect the viability of neuroblastoma cells up to 5 µg/mL [12].

Lahiri et al. studied the behavior of osteoblast cells in a scaffold constructed from the BNNT-embedded polylactide-polycaprolactone (PLC–BNNT) in orthopedic implants [75]. Using real-time PCR methods, they studied the *RunX2* gene expression profile, which is a transcription factor responsible for enhancing the cell proliferation. The results of the experiments showed that the PLC–BNNTs increased the *RunX2* gene expression of the osteoblast cells up to sevenfold. It was concluded that the positive effect of the BNNTs embedded into the scaffold on the cell proliferation was due to the natural affinity of proteins to the hydrophobic BNNTs [75].

Ciofani et al. investigated the cytotoxicity of GC–BNNTs [74]. The production of reactive oxygen species (ROS), DNA content in cell lysates, and apoptosis of cells were assessed using SH-SY5Y cells. The cells were exposed to GC–BNNTs up to 100 µg/mL. They found that the GC–BNNT-dependent toxic concentration was lower than the 50 µg/mL. On the other hand, 100 µg/mL of GC–BNNTs significantly decreased the cell viability. It was also found that the ROS production was not significant [74].

The hemolytic and cytotoxic effects of pure BNNTs on the malignant U87 (wild type p53), T98 (mutant p53) glioblastoma, MCF-7 adenocarcinoma mammary gland cells and normal MRC-5 fibroblast lung cells were investigated [76]. The hemolytic activity of the pure BNNTs was investigated by UV–vis spectroscopy and the results showed no significant hemolysis of cells that were exposed to the BNNTs. The metabolic activity of the BNNT-exposed cells using an MTT assay was studied and found that the BNNTs were significantly toxic at 200 µg/mL. The biocompatibility tests indicated that the pure BNNTs were good candidates at nontoxic concentrations for pharmacological applications [76].

The cell lines A549, RAW264.7, 3T3-L1 and HEK293 were exposed to BNNTs. The authors studied the cells with MTT and fluorometric microculture cytotoxicity assay (FMCA). As an indirect cytotoxicity measurement technique, FMCA assesses the esterase activity of cells. In general, the results indicated that the BNNTs were cytotoxic for the studied cell types even at low concentrations. In addition, the authors evaluated the toxicity of BNNTs according to the cell type and endocytosis ability of the cells. In particular, RAW 264.7 had high endocytosis capability as compared to A549 and 3T3-L1 cell types, and HEK293 showed low endocytosis capability. The results showed that the BNNTs slightly affected the growth and metabolic activity of HEK293 cells [77]. On the other hand, the effect of the BNNTs on A549 and 3T3-L1 cell types was worse than that of the HEK293 cells. The BNNTs were highly toxic for the RAW 264.7 cell type. From this study, one can be conclude that the toxicity of BNNTs is related to the cell type and the ability to perform endocytosis [77].

The morphology and viability of the GC–BNNTs-exposed HUVECs cells were also investigated [71]. The cells were incubated at increasing concentrations of GC–BNNTs for 48 and 72 h. The cell morphology and cell viability by amido black assay and trypan blue were studied. The total protein content and E1/1 protein expression profile were determined. It was found that a 50 µg/mL concentration of GC–BNNTs added to the cell medium caused a decreased proliferation. On the other hand, a 100 µg/mL concentration of GC–BNNTs showed a modestly reduced proliferation at hours 48 and 72. Similar to the results reported by Ciofani et al., the GC–BNNTs were nontoxic at low concentrations [71].

The toxicity of glucosamine (GA)-, poly(ethylene glycol)_1000_ (PEG_1000_)- and chitosan (CH)-coated BNNTs using MRC-5 cells was studied [78]. The study found that the BNNT–CH and BNNT–PEG were nontoxic in the range from 0.1 to 50.0 µg/mL but they were significantly toxic at 100 µg/mL. It was concluded that the GA–BNNT, PEG–BNNT and CH–BNNT were nontoxic at low concentrations but further evaluation on more cell types was suggested for their reliable use in biomedical applications [78]. The GA–BNNTs were found to be nontoxic to MRC-5 cells, but the previous studies claimed that glucosamine prevented the cellular uptake of glucose by pancreatic beta cells and caused cell death [73].

Poly-L-lysine (PLL) is known for its cytocompatibility due to the presence of amino groups and its use as a good dispersion agent [79]. The BNNTs were first coated with PLL and then with quantum dots (QDs) to observe the cellular uptake of PLL–BNNTs in C2C12 mouse myoblast cells. To observe the energy dependence of the uptake mechanism of the PLL–BNNTs, sodium azide was used to block ATP. They concluded that the PLL–BNNTs accumulation occurred in the cell membrane with energy dependent pathways. At a concentration of up to 10 µg/mL, the PLL–BNNTs exhibited no evidence of apoptosis, necrosis and membrane permeabilization [79].

Danti et al. investigated the cellular uptake of the BNNTs in the primary human osteoblasts (hOBs) under the exposure of low frequency ultrasound (US) [80]. The BNNTs were wrapped with PLL for stabilization in water. Furthermore, they found that the PLL–BNNTs were localized in the cytoplasm in the vesicles. Although the experimental system was complex, and further studies were necessary to understand the molecular mechanism, an obvious result was the increasing osteoblastic maturation. The findings of this study indicated that the BNNTs had potential use as nanotransducers for cellular therapies [80].

Ciofani et al. first reported a pilot investigation on the in vivo toxicity of BNNTs and they applied a single intravenous dose of BNNTs at 1 mg/mL. The changes in blood, kidneys and liver parameters were observed [81]. In the following study, 5 and 10 mg/kg of the BNNTs were intravenously injected into rabbits [82]. After the injection, the white blood cells, red blood cells and hematocrit levels were analyzed to investigate the possible adverse effects of the BNNTs. The authors did not see any adverse toxicological effect of BNNTs in blood [82].

Some of the important studies from the literature are summarized above and some of the toxicity assessment attempts are provided in Table 2. As seen, a number of reports claim that the BNNTs are nontoxic. Since the BNNTs are highly hydrophobic, it is difficult to perform toxicity assays for these materials. Therefore, a surface modification approach is generally performed to increase their dispersibility in aqueous media (Table 2). However, this may mask the real toxicity of the BNNTs since mostly the surface coating comes in contact with the cells. Based on the reports to date, their toxicity depends on the concentration, cell type and surface modifications, as is the case for all nanomaterials. A possible reason for the disagreement among the reports could arise from the synthesis procedure of the BNNTs, since the chemicals for the synthesis vary from synthesis to synthesis. Therefore, a careful purification step is vital for the further use of the BNNTs after the synthesis. Finally, although all in vitro studies provide very valuable data for the toxicity assessment, evaluation of this novel material under in vivo conditions is critically important. At the moment, a lack of in vivo data is one reason a solid conclusion about the toxicity of the BNNTs cannot be drawn.

**Table 2:** Toxicity behavior of the BNNTs on cultured cells and animals.

Physical coating	In vitro / In vivo	Type of assay	Result	Ref.

PEI	SH-SY5Y	Trypan Blue, MTT	Nontoxic at 5 µg/mL	[12]
glycol chitosan	SH-SY5Y	MTT, WST-1, Apo. kit, Image-IT Green ROS kit	Low toxicity <100 µg/mL	[74]
PLC	Osteoblast cells (*RunX2*)	Real-time PCR	Increased cell growth	[75]
nonfunctionalized	U87, T98, MCF-7, MRC-5	MTT	Low toxicity <200 µg/mL	[76]
nonfunctionalized	A549, RAW264.7, 3T3-L1, HEK293	MTT, FMCA	Related to cell type	[77]
glycol chitosan	HUVECs	Amido Black assay, Trypan Blue	Nontoxic <50 µg/mL	[71]
glucosamine, PEG, chitosan	MRC-5	MTT	Nontoxic <50 µg/mL	[78]
PLL	C2C12	Trypan Blue, MTT, LIVE/DEAD, annexin V-FITC	Low toxicity <10 µg/mL	[79]
PLL	hOB	MTT	Low toxicity <10 µg/mL	[80]
glycol chitosan	Rabbit	Blood tests	Nontoxic	[81–82]

### Drug delivery

The use of nanomaterials in drug delivery has also been investigated in recent years. Although the BNNTs can potentially be used for drug delivery, their behavior in biological environment is not very well understood at the moment. However, there are a few early reports and a review is included here.

A multifunctional, BNNT-based nanocarrier was synthesized using an Fe catalyst to render them magnetic and coated with PEI whereby QDs were attached to the PEI-coated BNNTs [83]. The cellular uptake of the BNNTs was tracked by QDs which revealed that the cellular uptake of the BNNTs depends upon the distance from the external magnetic field. A superconducting quantum interference device (SQUID) magnetometer analysis was carried out and revealed that the magnetic properties of the BNNTs were related to the Fe catalysts. Considering the magnetic properties and the ability to bind molecules on a large surface area of the BNNTs, it was concluded that the constructed structure was ideal for drug delivery purposes [83].

The core–shell structures of BNNTs with europium-doped, sodium gadolinium fluoride (NaGdF_4_:Eu) were fabricated by using urea to demonstrate the chemotherapy efficiency of the BNNT–NaGdF_4_:Eu composites in the presence and absence of a magnetic field [84]. The BNNT–NaGdF_4_:Eu composites simultaneously show fluorescent and magnetic properties. Thus, imaging and targeting of the composites can be more easily achieved. Human LNCaP prostate cancer cells were treated with the BNNT–NaGdF_4_:Eu composites in the presence and absence of a magnetic field and higher cell-associated uptake was found in the presence of a magnetic field. Then, the composites were loaded with doxorubicin (dox) to investigate the viability of LNCaP prostate cancer cells in the magnetic field. It was found that dox-loaded BNNT–NaGdF_4_:Eu composites had higher toxicity in the presence of a magnetic field due to increased cellular uptake of the composites and thus increased doxorubicin delivery. It can be said that the BNNT–NaGdF_4_:Eu composites increase the chemotherapy efficiency by the use of an external magnetic field [84].

The BNNT–mesoporous silica (BNNT–MS) hybrids were reported for their use in drug delivery [85]. In this study, a negatively and positively charged BNNT–mesoporous silica, BNNT–MS and BNNT–MS–NH_2,_ respectively, were produced. These structures were loaded with dox. The loading efficiency was found to be pH dependent and both negatively and positively charged structures had the same dox loading capacity. The BNNT–MS–NH_2_ had higher uptake potential in LNCaP prostate cancer cells due to its charge. Thus, it had a higher toxicity towards LNCaP prostate cancer cells. It was concluded that the prepared structures have potential in cancer therapy [85].

**Figure 6:**
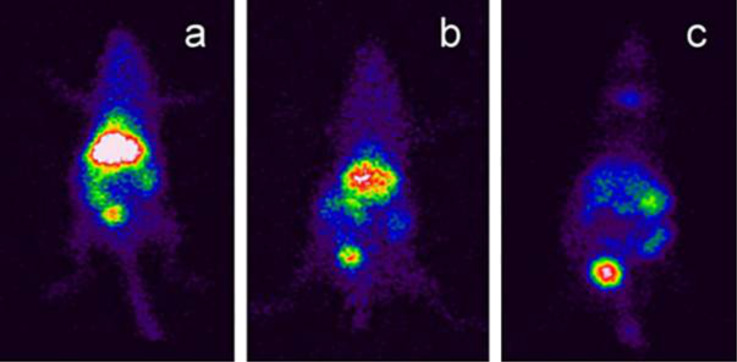
Scintigraphic image of radiolabeled, glycol chitosan BNNTs after (a) 30 min, (b) 1 h, and (c) 4 h after injection. Figure adapted with permission from [86], copyright 2012 Elsevier.

To investigate in vivo biodistribution of the BNNTs, they were functionalized with GC then radiolabeled with ^99m^Tc [86]. After 30 min of injection into mice, the BNNTs were in the systemic circulation and accumulated in the liver, spleen and intestinal tissues. After 4 h, radiolabeled BNNTs were found in the bladder. They were eliminated from body by renal excretion and the BNNTs conjugated with chitosan were degraded through enzymatic degradation. After 24 h, there was a reduction of radiation in the related organs (Figure 6). These promising results showed that the BNNTs have potential to carry new drugs or radioisotopes [86].

Electroporation is used for increasing the cell permeability for introduction of molecules into cells. However, it requires a high voltage, which is one of the problems for drug delivery. The applicability of BNNTs in an electroporation process was investigated [87]. The BNNTs were stabilized in phosphate buffered solution (PBS) with the help of PLL. Before applying electroporation to human neuroblastoma SH-SY5Y cells, the cells were incubated in the BNNT suspension, and then the electroporation was performed. The results demonstrated that a low electric field was adequate for electroporation. The BNNTs acted as mediators for electroporation as they interacted with the cell membrane. These experimental findings indicated that the BNNTs are promising tools for drug and gene delivery using electroporation [87].

A theoretical study revealed that platinum-based anticancer drugs preferentially interacted with Al-doped BNNTs as compared to pristine, zigzag and armchair BNNTs [88]. Cisplatin (cis-Pt) and nedaplatin (neda-Pt) molecules were used as platinum-based anticancer drugs. An aluminum (Al) atom is substituted for a boron atom. The Al atom induced a protrusion out of the plane of the BNNT and a distortion occurred at the doping site to relieve the stress. Density functional theory was performed to observe the absorption of cis-Pt and neda-Pt on pristine and Al-doped BNNTs. The results indicated that the chlorine atom of cis-Pt and the oxygen atom of the carbonyl group of neda-Pt interacted with the Al-doped BNNTs [88].

### Biomaterial applications

The addition of BNNTs into a polymeric matrix could increase the physical strength, degradation rate and durability of the final product. Therefore, their use in polymeric biomaterials was investigated.

**Figure 7:**
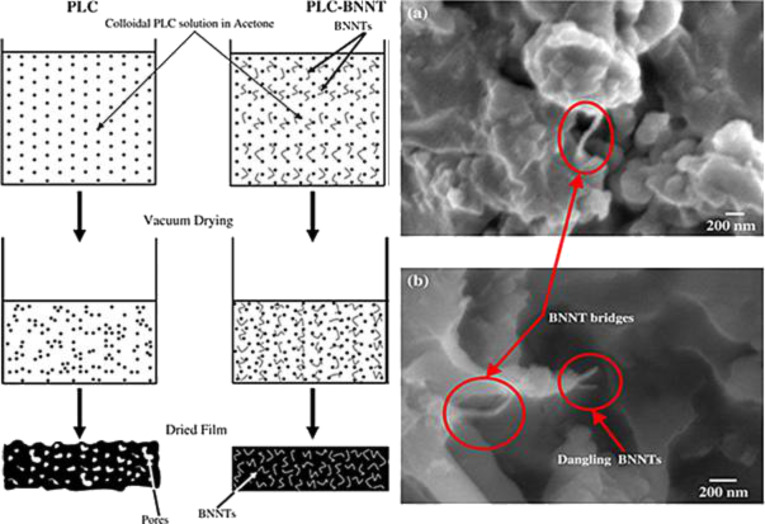
Preparation process of PLC (left) and PLC–BNNTs (right) and (a,b) SEM images of a PLC–BNNT composite exhibiting improved mechanical properties due to BNNTs bridges (red). Figure adapted with permission from [75], copyright 2010 Acta Biomaterialia.

In one study, the BNNTs were used in polylactide-polycaprolactone (PLC) copolymer as additives to improve the properties of the polymer as an orthopedic implant [75]. With the addition of BNNTs, a 1370% increase in the mechanical strength of the polymer was observed. The reason for such an improvement was attributed to the formation of BNNT bridges among the polymeric structures. Figure 7 shows such structures as marked in red. When the osteoblast cell viability was evaluated with respect to the BNNT–PLC composite, and compared only to PLC, an increased cell viability was observed. In addition, it was found that the *RunX2* gene, which is the major regulator for the differentiation of the osteoblasts cells, increased the expression level with the addition of the BNNTs into the polymer. These results indicated that BNNTs plays an important role to improve mechanical properties of a scaffold and up regulated the gene expression for increased cell viability for orthopedic applications [75].

Hydroxypatite (HA) is an important material used in orthopedic implant applications [89]. 4 wt % of BNNTs were added into HA to improve its mechanical properties. It was found that compared to HA alone, an increase in elasticity of up to 120%, a 129% increase in hardness, and an 86% increase in fracture toughness were possible. The BNNT–HA composite also showed a 75% increase in the wear resistance. It was noted that the addition of BNNTs to HA did not have any adverse effect on osteoblasts cell proliferation and viability [89].

### Sensing applications

One interesting application area of BNNTs is the field of sensors. Although there are not many reports regarding their use in sensors, a few available reports are included here as examples. The unique properties of the BNNTs can be combined with the properties of other nanomaterials to construct novel sensor devices for humidity, carbon dioxide detection, and clinical diagnostics.

**Figure 8:**
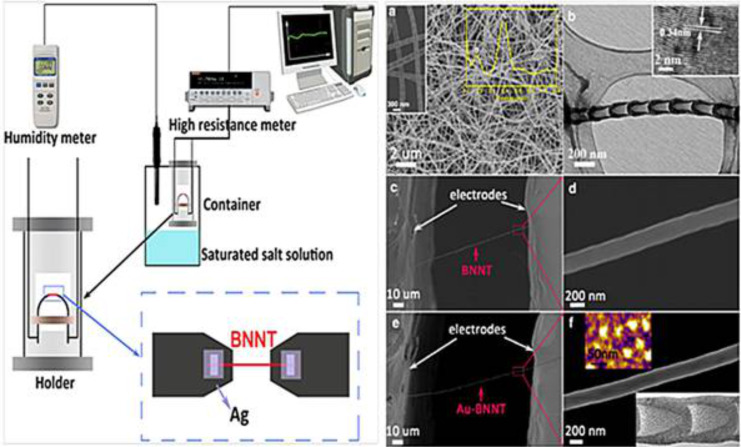
Schematic representation of a humidity sensor test system with a single BNNT and a single BNNT–AgNPs. (a) SEM image (left) and EDS spectrum (right) and (b) TEM and HRTEM image of the BNNTs, (c) and (e) the SEM images with a single BNNT and single AgNP–BNNT, (d) and (f) the higher magnification SEM images in (c) and (e) marked with red square (f) AFM (upper) and TEM (lower) images. Figure adapted with permission from [90], copyright 2013 Elsevier.

A highly sensitive humidity sensor using BNNTs and silver nanoparticles (AgNPs) for the rapid detection of humidity was fabricated [90]. Figure 8 shows the humidity sensor system. The adsorption–desorption tests showed that the AgNP–BNNT material had a potentially fast response/recovery time of 100/15 s for the detection of relative humidity at room temperature [90].

The use of Ni-encapsulated BNNTs in optomagnetic-based sensors with respect to their magnetic and optical band gap properties was evaluated [91]. Two intense blue emission peaks at ~480 nm and ~365 nm were observed upon encapsulation of BNNTs with Ni. The time-resolved photoluminescence spectroscopy (TRPL) provided a photoluminescence spectrum with a bi-exponential decay of 280 ps. It was suggested that the Ni-encapsulated BNNTs could be used in clinical diagnostics and bioimaging applications due to their TRPL properties [91].

### Hydrogen storage in BNNTs

Hydrogen is considered to be an exceptional energy source material since it produces clean energy in high yield. Although there are several techniques that can generate an abundant amount of hydrogen, its storage and transportation is an obstacle for its widespread use. In this respect, BNNTs were also investigated for their hydrogen adsorption capacity. Molecular dynamics simulations indicated that the collision and adsorption behavior of hydrogen molecules in single-walled BNNTs varies according to hydrogen incident energy. Additionally, the theoretical investigations showed that the B–N bond length (1.46 Å) of the BNNTs was larger than the C–C bond length of CNTs (1.42 Å). Therefore, the penetration of hydrogen molecules through the BNNT walls is faster than that through the CNT walls [92]. On the other hand, the B–N heteropolar bonding structure of the BNNTs induced an extra dipole moment on the hydrogen molecules contrary to the CNTs [34]. In addition, there are experimental reports supporting the simulation studies [11]. For the BNNTs, which were synthesized with Fe^3+^/MCM-41 (mobile composition of matter) complex catalysts, the percentage of the adsorbed hydrogen molecules was two times larger than for the commercial CNTs. The adsorption capacity of hydrogen molecules by BNNTs was measured as 0.85 wt % by the Intelligent Gravimetric Analyser at room temperature [11].

Margulis at al. theoretically investigated the preferable adsorption sites on the BNNT surface by using the semi-empirical AM1 method [93]. The computational models showed that the hydrogen atoms favorably bonded to the nitrogen atoms as compared to boron atoms due to the higher electronegativity of the nitrogen atoms. Because of the high electronegativity of the nitrogen atoms, the hydrogen atoms can come into closer contact than the boron atoms can, but with the same energy. The simulations indicated that the diameter was an important property of the BNNTs to increase the amount of stored hydrogen molecules [93].

The binding positions of the hydrogen molecules on the surface of the SWBNNTs were also investigated [94]. It was claimed that the hydrogen molecules had a capacity to bind to the SWBNNTs as perpendicular, longitudinal and transversal positions in an ab initio theoretical study. The hydrogen molecules approached the SWBNNTs in perpendicular position to the surface, which slightly polarized and raised the binding energy of the molecules. On the other hand, the hydrogen atoms interacted with the nitrogen atoms more than the boron atoms [94].

The number of walls and the diameter of each wall affect the hydrogen molecule storing capacity of the BNNT. The hydrogen physisorption capacity of the SWBNNTs and MWBNNTs was theoretically investigated by grand canonical Monte Carlo theoretical studies [95]. The simulations showed that the triple-walled (TW) BNNTs provided the necessary limited spaces for hydrogen atoms between the tube walls. The double-walled (DW) BNNTs were predicted to store more hydrogen atoms due to the large space within the nanotubes as compared to the TWBNNTs with the condition that the inner tube diameter was sufficiently large in the DWBNNTs. As a conclusion, the number of BNNT walls was found to be very important as well as the wall diameter, which could be chosen to inhibit the hydrogen–hydrogen repulsion in the inner space of the BNNTs for improved hydrogen atom storage capability [95].

The hydrogen molecule storage capacity for BNNTs of several morphologies were also investigated experimentally [96]. The studies showed that the number of absorbed hydrogen molecules increased with the hydrogen pressure. The flower-type, straight-walled and bamboo-type BNNTs were evaluated for their hydrogen adsorption capacity. It was found that the adsorption of the flower-type was 2.5 wt % at about 100 bar hydrogen pressure. The straight-walled BNNTs exhibited an increased hydrogen storage capacity up to 2.7 wt % and the bamboo-type BNNTs had the highest hydrogen uptake capacity of 3.0 wt % [96].

The BNNTs were synthesized using annealing and ball milling methods and the hydrogen storage on the BNNTs was investigated experimentally by pressure–composition isotherms (PCI) and temperature-programmed desorption (TPD) methods [34]. The results showed that the hydrogen uptake capacity of purified BNNTs was 2.2 wt %. The temperature effect on the hydrogen storage capacity of the BNNTs was also investigated in the study. The BNNTs were exposed to the hydrogen atoms at 180 and 250 °C and with resulting hydrogen absorption in the range of 1.6–1.2 wt % [34].

The theoretical investigation of the hydrogen storage capacity of BNNTs showed that the nature of the electronic structure of boron and nitrogen atoms, as well as the diameter and dimensions of the BNNT walls have an impact on their hydrogen adsorption capacity. Although there are limited numbers of reports, the studies show that BNNTs are possibly valuable materials for hydrogen storage. It is important to note that further experimental investigations addressing the parameters such as temperature, gas pressure, and purity of the BNNTs should be conducted.

### BNNTs for neutron capture therapy

**Figure 9:**
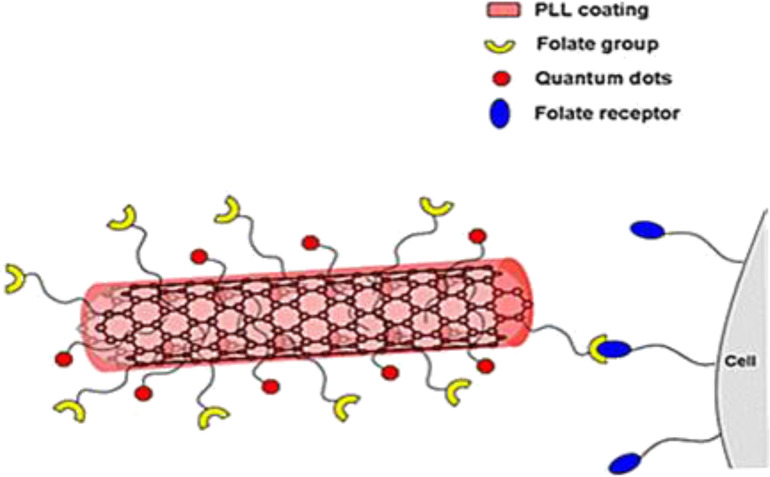
Schematic representation of a poly-L-lysine-, fluorescent probe- and folate-modified BNNT. Figure adapted with permission from the authors [97].

The neutron absorption capacity of BNNTs was also reported [97]. Ciofani at al. investigated the use of BNNTs as contrast agents for neutron capture therapy, which could be an innovative approach for treatment of several aggressive cancers such as cerebral glioblastoma multiform. The main purpose of the therapy was to target the tumor cells by ^10^B atoms. Accordingly, BNNTs were used as carriers of boron atoms. Figure 9 shows how the BNNTs were functionalized with PLL, a fluorescent probe (quantum dots) and folic acids. PLLs were wrapped around the BNNTs to induce a hydrophilic property. In addition, the BNNTs were coated with folic acids for selective interaction with the tumor cells. The malignant glioblastoma cells were exposed to functionalized BNNTs under in vitro conditions. It was observed that the PLL–F–BNNTs were effectively taken up by the malignant glioblastoma cells. This study suggests that the use of BNNTs should be further investigated for neutron capture therapy [97].

Gadolinium-doped BNNTs were fabricated as an effective contrast agent in clinical applications of BNNTs [98]. Due to the high magnetic moment property of gadolinium, Gd-doped BNNTs can be applied as an MRI contrast agent. The high in vitro biocompatibility property of the Gd-doped BNNTs and the labeling of cell populations due to the Gd and B content, make these structures a novel negative contrast agent [98].

### Conclusion

It is clear that there is an increasing trend for the application of BNNTs in several fields from medicine to sensors. Although their synthesis is rather straightforward, it is still not possible to produce large quantities with high purity and uniformity. Since their physicochemical properties are independent of chirality, a simple synthesis method can be suitable, in contrast to CNTs. However, the choice of boron and nitrogen precursor catalysts can be important factors for their possible medical and biomedical applications. The other important point is the cost of the preparation of BNNTs. In this sense, there is an effort to reduce the synthesis temperature. However, to date, it seems that a reasonably high temperature of around 1000 °C is currently required for synthesis. The mechanism of BNNT formation is mostly defined by the synthesis conditions, which should be better understood for the control of the experimental parameters. Another problem limiting their applicability is their low dispersibility in aqueous media due to their high hydrophobicity. In order to increase their hydrophilicity, either covalent modifications or physical adsorption of a molecule or polymer is typically performed. Covalent modification can be achieved through –OH groups on B atoms and –NH_2_ groups converted from N atoms at the edges and at defects.

The toxicity issues appear partially resolved in recent reports after conflicting early reports. However, there is a lack of in vivo data for the complete understanding of their toxicity. Most of the toxicity studies reported to date involve a polymer or a molecule attached to the BNNT surfaces to increase the dispersion in aqueous media, which might give misleading results since toxicity is mostly defined by the coating material. In addition, there is not much information about the fate of these materials in biological systems and the environment, such as their degradation after their release.
